# A framework for conceptualizing dimensions of social organization in mammals

**DOI:** 10.1002/ece3.5936

**Published:** 2019-12-16

**Authors:** Lea Prox, Damien Farine

**Affiliations:** ^1^ Department of Biology University of Konstanz Konstanz Germany; ^2^ Department of Sociobiology/Anthropology University of Göttingen Göttingen Germany; ^3^ Behavioral Ecology & Sociobiology Unit German Primate Center Göttingen Germany; ^4^ Department of Collective Behaviour Max Planck Institute for Animal Behavior Konstanz Germany; ^5^ Center for the Advanced Study of Collective Behaviour University of Konstanz Konstanz Germany

**Keywords:** framework, mammals, primary unit, social behavior, social organization

## Abstract

Mammalian societies represent many different types of social systems. While some aspects of social systems have been extensively studied, there is little consensus on how to conceptualize social organization across species. Here, we present a framework describing eight dimensions of social organization to capture its diversity across mammalian societies. The framework uses simple information that is clearly separated from the three other aspects of social systems: social structure, care system, and mating system. By applying our framework across 208 species of all mammalian taxa, we find a rich multidimensional landscape of social organization. Correlation analysis reveals that the dimensions have relatively high independence, suggesting that social systems are able to evolve different aspects of social behavior without being tied to particular traits. Applying a clustering algorithm allows us to identify the relative importance of key dimensions on patterns of social organization. Finally, mapping mating system onto these clusters shows that social organization represents a distinct aspect of social systems. In the future, this framework will aid reporting on important aspects of natural history in species and facilitate comparative analyses, which ultimately will provide the ability to generate new insights into the primary drivers of social patterns and evolution of sociality.

## Graphical abstract

Social organization is an aspect of animal social systems that has not been conceptualized yet and is often reduced to group size. This framework describes eight central dimensions of social organization to capture its diversity across mammalian societies. By applying our framework across 208 species of all mammalian taxa, we find a rich multidimensional landscape of social organization.

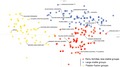

## 1 INTRODUCTION

Sociality is widespread in mammals and can take many different forms. Most mammals are social at least during the period of reproduction and parental care. However, sociality often extends beyond the reproductive period (Clutton‐Brock, 2016), with individuals living in groups, that is, sharing the spatiotemporal environment with conspecifics and forming social bonds with group members that have extensive impact on their, and their offspring's, survival (Silk, 2007; Silk, Alberts, & Altmann, 2003). Different terms and systems of classification have been proposed to capture the different forms of societies that animals are found in. Currently, most agreement exists in how to classify mating systems (e.g., monogamy, polygyny) (Clutton‐Brock, 1989; Reynolds, 1996). However, social systems are likely to represent the outcome of many different types of behavioral mechanisms that are not wholly centered on mating behavior. One potentially important aspect of sociality is social organization, defined as the size and composition of a social unit (Jarman, 1974, see Box 1). Developing a better understanding of the variation in social organization independently from those pertaining to the other aspects of social systems (social structure, mating systems, care systems (Kappeler, 2018)) could provide important information about the evolution of mammalian social systems. A framework that captures distinct measures of social organization could function as a base for comparative studies that investigate contexts between sociality and aspects as cognitive abilities or ecological drivers by pointing out relevant features that discriminate societies.

In previous approaches to resolve the challenge of comparing social systems across taxa, several publications have tried to streamline terminology (Clutton‐Brock & Janson, 2012; Nonacs & Hager, 2011). However, most studies exploring interspecific variation in social systems have been limited to a few social measures, such as group size (Dunbar, 1998; Pérez‐Barbería, Shultz, & Dunbar, 2007), that do not necessarily stand in direct relation with, for example, social complexity (Bijl, Buechel, Kotrschal, & Kolm, 2018; Kverková et al., 2018). Those studies that have tried to synthesize variation in broader social categorizations have focused on single lineages, such as in primates (Lee & Lee, 2001; Shultz, Opie, & Atkinson, 2011; Smuts, Cheney, Seyfarth, & Wrangham, 2008), cetaceans (Connor, Mann, Tyack, & Whitehead, 1998; Michaud, 2005), rodents (Wolff & Sherman, 2008), or bats (Kerth, 2008), or on specific aspects of sociality, such as mating systems (Clutton‐Brock, 1989; Reynolds, 1996), social predation (Lang & Farine, 2017), or care systems (cooperative breeding) (Bergmüller, Johnstone, Russell, & Bshary, 2007). What is needed are quantifiable metrics to describe different aspects of social behavior. Social patterns, such as those described above, can be represented as independent dimensions and given a score along a scale. Having clear definitions with corresponding numerical scores can then allow new insights about how dimensions relate to one another. For example, sets of scores can be analyzed using cluster analyses that identify similarities and differences in patterns of behaviors across species. Previously developed concepts can be mapped onto the cluster landscape (Lang & Farine, 2017), which can bring new ideas into established ways of thinking.

**Figure 1:**
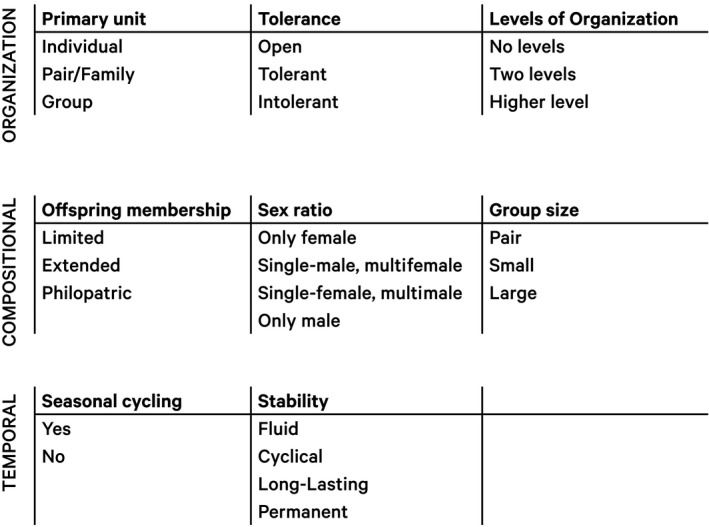
Scheme of framework. The eight dimensions can be broadly classed into three categories (organizational, composition, and temporal). Each dimension is made up of different categories that are given a distinct score (see text)

We propose a framework that describes aspects of mammalian social systems outside of the reproductive context, focusing on social organization. Without intending to neglect the importance of mating systems, that cause or are caused by social organization (Kappeler & Schaik, 2002; Sussman & Garber, 1987), we believe that looking beyond reproductive behaviors offers valuable opportunities to gain broader insights into the foundations of animal societies, including if and how mating systems map onto dimensions among broader aspects of sociality. We base the foundations of our framework on a paper by Peter Kappeler that looks at constraints and flexibility in mammalian sociality (Kappeler, Barrett, Blumstein, & Clutton‐Brock 2013). Here, the authors split up sociality into three aspects: social organization, social structure, and mating systems. Social organization has previously been used as the term encompassing both social structure, mating system, and various other social aspects of a social system (Baird & Whitehead, 2000; Smith, 1968; Tyler, 1972). It is not unusual that studies use the term synonymously with “social structure” which describes the patterns arising from associations and interactions between individuals (Whitehead, 2008). Here, we use the definition of social organization as pertaining to group size and composition (Jarman, 1974; Kappeler, 2018) (see Figure 1). In their 2013 paper, Kappeler et al. argued that for social organization, animals have basically three options: to live solitarily, to coordinate their activities with a partner, or to coordinate them with a whole group. Using our framework, we will show that there is possibly a fourth option alongside with those suggested.

We have identified eight distinct dimensions that can be used to describe different features of social organization in nonsolitary mammalian societies. These dimensions emerged from extensively reviewing literature and gathering input from researchers studying various taxonomic groups of mammals. The number eight was a result of our goal to find dimensions which reflect behaviors that should be simple to quantify with general knowledge of a species' natural history, are largely independent of each other, and captured the elements of societies that were not contained within aspects of social systems. We intend the framework to be used on different components of societies (later referred to as society components) that are persistent in their social organization, which could represent different parts of the population of a given species (e.g., different sexes or different populations that have contrasting social behaviors). Our framework can therefore investigate species that show intraspecific variation in their social systems (Schradin, Hayes, Pillay, & Bertelsmeier, 2018). Examples include those where males and females live separately outside of the breeding season, as in elephants and many ungulate species (Coulson, Albon, Guinness, Pemberton, & Clutton‐Brock, 1997; Poole, 1989), or where sexes are organized in different ways, as in lions where one or multiple males' territory might encompass several cohesive female prides (Kleiman & Eisenberg, 1973). Each dimension in the framework represents a unique and independent aspect of mammalian social organization, for which a society component can be scored in a way that is unambiguous given our descriptions. Using the dimensions, we investigate which are responsible for most differences within social organization. We hypothesize that social organization is mostly predetermined by the smallest stable unit (which we call the primary unit) and the sex ratio of the society. We then also examine the link between social organization and mating systems. While mating systems have an unquestioned impact on social systems in general, we hypothesize that they do not explain all differences observed in social organization.

## 2 THE FRAMEWORK

Below are a description and details for how to score each of the eight dimensions in our framework (Figure 1). Scores can be given to all individuals in a species, or separately to different populations or sexes (society components) if these differ in their social organization. If different populations or sexes of the same species would have been ranked differently within the framework, we ranked them separately. We used a method that was not sensitive to the absolute scale, scaling dimensions as 0 for when a feature was absent and 1 for the lowest level of it.

### Primary unit

The primary unit is defined as the largest stable unit that has a temporally consistent membership. The unit size is defined as the level at which any social change in the composition is permanent (i.e., does not change back). The importance of the concept of permanent change in this dimension can be illustrated by comparing Atlantic spotted dolphins with mantled howler monkeys (Aguilar‐Melo et al., 2013; Elliser & Herzing, 2014). In both species, groups frequently fission and fusion. Atlantic spotted dolphins come together in groups with membership being drawn from an open pool of individuals. When groups of howler monkeys split into subgroups for foraging, these can also have different compositions from one time to the next, but are drawn from a closed pool of individuals. If a howler monkey starts associating with individuals from outside this group (disperse), it is unlikely to return back into its original group. These examples represent the difference between a primary unit for the dolphins of “individual” and a primary unit for the howler monkeys of “group.”

#### 1 – Individual

Individuals change their social environment frequently. This results in unpredictable group compositions (in terms of identity of those in the group) and thus fluid association patterns. They could, for example, live in what van Schaik (1999) described as “individual‐based fission–fusion” societies. This is common in ungulates, such as chitals and cape buffalos (Asensio, Korstjens, & Aureli, 2009; Le Hellaye, Goossens, Jamart, & Curtis, 2010; Ramos‐Fernández & Morales, 2014), and also observed in many dolphin species, such as the Atlantic spotted dolphin mentioned above (Karczmarski, Würsig, Gailey, Larson, & Vanderlip, 2005; Lusseau et al., 2006; Pearson, 2009).

#### 2 – Pair or family

Pairs of individuals persist over time and do not separate without forming new pairs (but can, e.g., forage separately). Examples include jackals (Moehlman, 1987) or species of lemurs (Wright, 1990). Families represent similar types of units to pairs, as they are small groups and the group is composed of mostly related individuals that have (at least in a single generation) come from the same genetic source or live in a matrilineal society. Species found in pairs or families include matriarchal groups of killer whales (Whitehead, 1998) or species that breed cooperatively such as meerkats (Young et al., 2006).

#### 3 – Group

A group is stable in a similar way as the previously described family, but usually larger and with offspring of one generation coming from different genetic sources. Those groups might fission into smaller subunits or fuse with other groups, but always maintain consistent membership within the unit.

### Tolerance

Tolerance refers to how primary units interact with other primary units and is rated on a scale from 0 to 2.

#### 0 – Open

Primary units can freely join other primary units. The lack of entry restriction (“free entry groups” (Ward & Webster, 2016)) is typical for large herds of ungulates such as African buffaloes (Focardi & Paveri‐Fontana, 1992) and can also be found in societies with high degree of fission–fusion. Aggression is rarely observed.

Box 1 **Glossary**

**Fission–fusion society**

The term “fission–fusion” was first introduced by Hans Kummer (1971) and originally describes “a society consisting of casual groups of variable size and composition, which form, break up and reform at frequent intervals” (Conradt & Roper, 2000). Societies that are classed as fission–fusion are typically found among the mammals and include species of primates (Asensio et al., 2009; Lehmann, Korstjens, & Dunbar, 2007), carnivores, such as hyenas (Smith, Memenis, & Holekamp, 2007), ungulates (Sundaresan, Fischhoff, Dushoff, & Rubenstein, 2007), and dolphins (Lusseau et al., 2006; Parra, Corkeron, & Arnold, 2011). More recently, the term has been as well used in association with other vertebrates such as fish and birds. Where the line between fission–fusion and cohesive societies should be drawn often depends on the interpretation of “frequent intervals.” Aureli proposed that fission–fusion should rather be seen as a gradient than a modal type of a social organization as it is present to less or more extent in far more societies than originally thought.

**Multilevel society**

“Multilevel” is a modal type of social organization that is used for societies that include multiple hierarchically nested levels. A typical example for this is hamadryas baboon societies where the highest level is the troop consisting of up to several hundred individuals, in which individuals aggregate at the same sleeping spots (Kummer, 1967; Schreier & Swedell, 2009). The troop is composed of multiple bands—stable units that coordinate their activities during the day and travel together. Within the band, the next lower level is the one‐male unit, a unit with one leading male and several females. Similar organizations can be found mostly in other primates (Grueter, Chapais, & Zinner, 2012), but also in African elephants (Archie, Moss, & Alberts, 2006), sperm whales, and some ungulates (Rubenstein & Hack, 2004).

**Social structure**

Social structure is the emerging property of the ways of interaction between individuals (Whitehead, 2008). A good context to base this on is by Robert Hinde, who developed a conceptual framework for the analysis of animal societies in 1976 (Hinde, 1976). This framework is built up of three levels, of which the uppermost is “surface structure,” the social structure as perceived by the observer. The two layers leading to this are interactions and the relationships that arise out of such successive interactions. Social structure emerges from relationships of individuals and determines them at the same time.

**Social organization**

While social structure exclusively looks at structural aspects of a society, social organization describes the size and composition of the social unit. This includes sex ratios, philopatry of offspring, and membership of the social unit in higher levels (age, sex, relatedness of group members).

**Social system**

The social patterns of a society.

**Society**

Set of individuals that interact with each other on different levels that can be made up of multiple society components, such as males and females with different social organizations in some cases. Societies can also include different species that associate with each other.

**Society component**

All individuals of a species that share consistent patterns of social organization and thus would be scored the same in all dimensions using this framework.

#### 2.1 Tolerant

Primary units can mix, but some low level of agonism or local avoidance can be observed. This is found, for example, in African elephants that from time to time form larger aggregations but quite clearly maintain their original groups within these aggregations (Archie et al., 2006).

#### 2.2 Intolerant

At any level of the society, territory or other resources are defended against other groups. Direct encounters of society components are usually associated with high levels of aggression or completely avoided. Examples include yellow mongooses (Le Roux, Cherry, & Manser, 2008) and Jamaican fruit bats (Morrison, 1979), where groups are defended against intruders.

### 2.1 Levels of organization

In some cases, the primary unit is one part of multiple levels of grouping. Primary units might temporarily associate with other groups within a broader social community, after which they split back into their original groups. Such societies are often called “multilevel societies” and are regarded as among the most complex. They have mostly been studied in primates but have also been described for other mammal species. The levels refer only to nestedness upward from the primary unit. If groups are nested within the primary unit, this would be referred to as social structure and therefore represents a different aspect of the social system.

#### 2.1.1 Unit level

Individuals, pairs, families, or groups that are not part of a higher level. This is true for many species that are territorial or where encounters are determined only by individuals' spatial distributions.

#### 2.1.2 Two levels

Primary units that are part of one higher level of organization. Often, this higher level is referred to as “community” or as “social unit” (Newman, 2006; Whitehead, 2008). Many fission–fusion societies with individuals as their primary unit can fall into this category. A classic example of a two‐level society is gelada baboons, where the primary units are either one‐male units (OMUs) with a number of females or all‐male units (AMUs), containing a number of bachelor males. Several OMUs and AMUs make up a band that travels together and shares common resources (Kawai, Ohsawa, Mori, & Dunbar, 1983).

#### 2.1.3 Multiple levels

A multilevel society exceeding two levels is categorized as multilevel society. A typical example for this is hamadryas baboons that form one‐male units within clans like the geladas, but their clans are additionally members of even larger bands (Schreier & Swedell, 2009). Other examples include killer whales with core groups, bond groups, and clan groups (Archie et al., 2006), or golden snub‐nosed monkeys (Qi, Li, Garber, Ji, & Watanabe, 2009).

### 2.2 Offspring membership (to primary unit)

The time offspring spend with their natal primary unit is central to group composition and, therefore, social organization. If generations of offspring overlap or if they are philopatric, it will affect the genetic relatedness of the group.

#### 2.2.1 None

No parental care is given during the time the society component is monitored. This usually applies to all‐male groups.

#### 2.2.2 Short/limited

The members of one generation of offspring disperse before or when a new generation of offspring is born. Offspring of polar foxes, for example, leave their parents in their first winter (Eide, Jepsen, & Prestrud, 2004).

#### 2.2.3 Extended

Generations of offspring overlap, or the next reproductive event is delayed until parental care for current offspring is completed. An example is Eastern Grey Kangaroos that simultaneously ween two offspring of different ages (Poole, 1975).

#### 2.2.4 Philopatric

The majority of offspring of the society component stays in the group after reaching maturity. This can be seen in matrilines, patrilines, or large groups. Examples include female South American Coatis (Hirsch, 2007) or white‐tailed deer (Lesage, Crête, Huot, Dumont, & Ouellet, 2000).

### 2.3 Sex ratios

This refers to the number of adult females and males present in a typical social context the individuals are observed in. Usually, this will be the primary unit, but for societies where the primary unit is an individual and that exhibit a high degree of fission–fusion, the sex ratio of a typical group is scored.

1 – Only males
2 – One female, multimale
3 – Multimale, multifemale
4 – Single‐male, multifemale
5 – Only females

### 2.4 Group size

The typically observed number of adults in the primary unit, or the size of a typical group when the primary unit is an individual. A natural classification occurs at group sizes of less than versus greater than or equal to 7, as the median value for group sizes in our dataset (see below) was 6.68. Further, it has been suggested in birds (Giardina, 2008) and baboons (Farine et al., 2016) that the number of conspecifics individuals are able to pay attention to is around 6.

#### 2.4.1 Pairs

In most cases, this refers to male–female pairs, but pairwise units can also be found among coalitions often consisting of two males as it happens for male alliances.

#### 2.4.2 Small groups

Six animals or fewer.

#### 2.4.3 Large groups

Seven animals or more.

### 2.5 Seasonal variation

Many species show seasonal variation in social organization, such as disbanding their groups to form pairs during the nonbreeding season (or vice versa).

#### 2.5.1 Yes

The society component has different patterns throughout the year and thus would be scored differently in at least one dimension using this framework if using data from different seasons. Examples for this are many ungulate species or animals that are solitary outside of the breeding season, such as female sperm whales (Whitehead & Kahn, 1992).

#### 2.5.2 No

The social patterns of the society component are independent of seasonal change and can be observed at all times of the year. These can fluctuate over time, but would be given the same score when using this framework across all seasons.

### 2.6 Temporal stability

This refers to the general stability of group membership. How likely is it to re‐observe two adults together after a certain time? Individuals from the same family or group (for primary units 2 and 3) might remain together all the time or frequently split into subgroups. In other cases, individuals might remain in cohesive social constellations, but these have a constant, albeit slow, turnover in membership.

#### 2.6.1 Fluid

Individuals are likely to be observed with different associates over the course of short time periods (e.g., hourly or daily), and groups change membership constantly over time. From one observation to the next, any changes in group composition would be unpredictable in terms of the number of individuals that have left or joined. Societies with the primary unit 1 will generally score 0 in this dimension. This is typical for societies with a high degree of fission–fusion, where individuals repeatedly move between groups such as nyalas that live in constantly changing groups (Anderson, 1980).

#### 2.6.2 Cyclical

Group memberships change with regular periodicity. This can be found in societies that have a strong seasonal pattern in their social organization, that is, from different types of societies during or outside the breeding season. Many ungulate species such as wapitis come together in multimale–multifemale groups for reproduction and then segregate into same‐sex groups afterward that might have slightly different compositions every time (Altmann, 1951).

#### 2.6.3 Long‐lasting

A social constellation is maintained over longer time periods. Individuals may have changed groups only a few times in their lifetime. Changes in membership can arise from demographic factors or changes in resource availability. In societies that are centered around a single dominant pair, for example, the loss of one of the alpha individuals could cause the remaining members to split up and join new constellations. Partner re‐emplacement can happen, for example, in giant otters when one of the alpha animals dies or disappears (Evangelista, 2004). In other cases, when groups get too large or the usual sex ratio is shifted strongly toward one or the other end, groups of individuals might split off, as in Thomas's Langurs (Sterck, 1997).

#### 2.6.4 Permanent

Individuals are not known to change groups during adulthood. Societies like this can be found in species that occupy large isolated territories where emigration is hindered by large distances, or groups that have stable membership that is closed to outsiders, as found in matrilines of female African elephants (Archie et al., 2006).

## 3 METHODS

### 3.1 Data collection

We used all species described in the first seven volumes of the Handbook of the Mammals of the World (HBMW) (Wilson, Mittermeier, & Lacher, 2019) from which we could extract sufficient data across the eight dimensions, a total of 208 species from 129 genera. These spanned primates, hoofed mammals, marine mammals, marsupials, rodents, and carnivores. To this, we added nine species of bats using information published in peer‐reviewed papers (Brooke, 1987; Dechmann, Kalko, König, & Kerth, 2005; Heckel & Von Helversen, 2003; Johnson, Kropczynski, Lacki, & Langlois, 2012; Kunz, August, & Burnett, 1983; Morrison, 1979; Park, Masters, & Altringham, 1998; Silvis, Kniowski, Gehrt, & Ford, 2014; Vonhof, Whitehead, & Fenton, 2004). We scored males and females separately if they had different scores across at least one dimension. The same applied to societal components with different scores in different seasons, but these were only scored outside the breeding season. We ran all analyses once with all species included and once restricting our data to one species per genus. We repeatedly tested the definitions in the framework by asking different people to evaluate example species and ensure they always gave the same scores.

### 3.2 Analysis

We first calculated the correlation among the species' scores across each pair of dimensions to determine the interdependence among variables. To identify sets of species (or components) with similar scores, we performed principal component analyses (PCAs) using the R packages *FactoMineR* and *factoextra* (Husson, Josse, Le, Mazet, & Husson, 2018; Kassambara & Mundt, 2016). The PCA was based on a covariance matrix to allow for weighted variables after scaling the scores from each dimension. As the variables chosen might have different importance on the social organization, the variables accounting for more variance in the outcome are given more weight and will therefore have a larger impact on the first component than a variable with a small weight. Variables with smaller loading might be an essential part of social organization itself, but not as deterministic as other variables. The resulting coordinates of the individual species were assigned to clusters using finite Gaussian mixture modeling (FGMM) implemented in the R package *mclust* (Fraley & Raftery, 1999). Finally, we plotted the different mating systems (following terms associated with the regarding species in HBMW (Wilson et al., 2019)) onto the dimension clustering.

We performed a DFA and decision tree analysis implemented in the program KNIME (Meinl et al., 2009) on a subset of 33 society components for which information was available across all dimensions to test whether these were consistently described by patterns of social organization. The tree‐like structure constitutes nodes that represent class labels and branches representing the possible decisions. The tree was trained using all society components with complete entries (i.e., which had information in all dimensions) and of which the mating system was known. The algorithm creates the tree in a way that increases the purity of the subsets regarding the target variable. We used the Gini index as a quality index. This index measures the impurity of a possible outcome. The feature with the outcome that has the lowest Gini index is then chosen by the learning node. By applying this method, the features (i.e., dimensions) which are able to create the outcome with highest purity are chosen to split the subset of data into groups.

## 4 RESULTS

**Figure 2:**
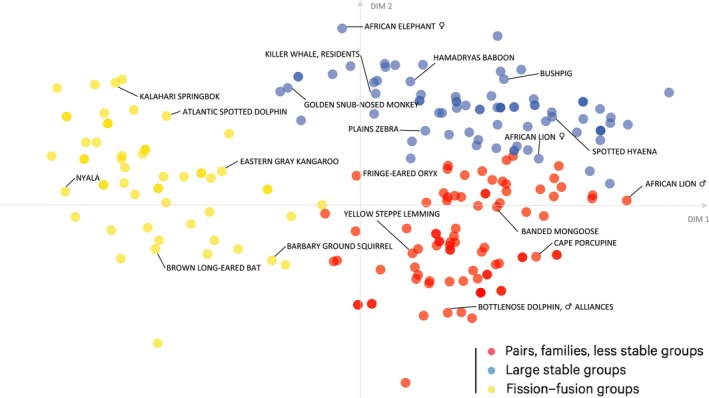
Distribution of society components across cluster landscape. After performing a PCA, the resulting coordinates of 220 society components across 208 species for each of the eight variables were reduced to two dimensions. Circles are colored according to the clusters they were assigned to by the FGMM. Society components within the yellow cluster are characterized by “individual” as primary unit, fluid stability, and mostly open tolerance. Society components within the blue cluster are mostly larger groups and families with long‐lasting to philopatric offspring membership and long‐lasting to permanent stability. The red cluster consists of society components that mostly live in pairs or families with offspring that are generally not philopatric (except for few exceptions). Society components in the red cluster tend to be more tolerant than those in the blue cluster. The results from the genus‐level analysis are given in Figure A3

**Figure A1:**
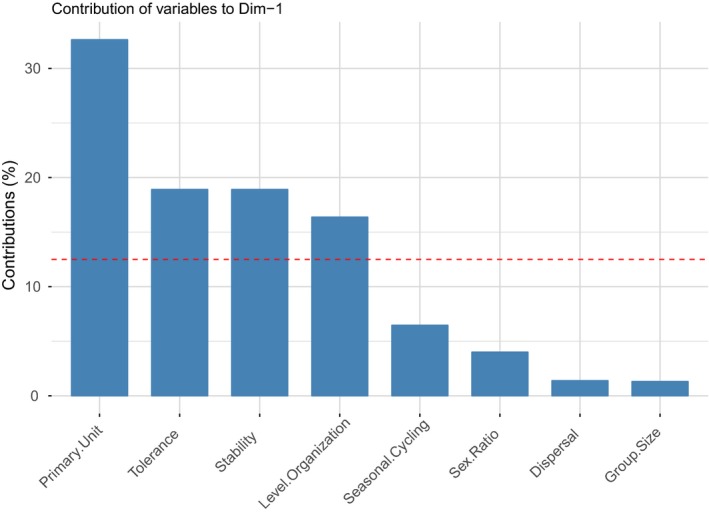
Contribution of variables to first principal component. The percentage to which each variable contributes to the first component after performing the PCA on the scores of the society components. The red dashed line indicates the expected average contribution. Contributions of first variables: primary unit: 32.55%; tolerance: 18.63%, stability: 18.61%; level organization: 6.53%

All results were largely consistent across genus‐level and species‐level analyses. Variables seemed to be fairly independent, with only primary unit and stability being strongly correlated (0.82 both species‐level, 0.84 genus‐level), suggesting that they all contributed unique information. The way the variables are clustered shows connections between them. When looking at the contributions of the different variables to the principal components, the first three components accounted for 67.74% of the variance (Figure A1). Primary unit had the highest loading (34.07% species‐level; 35.23% genus‐level) on the first component. Together with stability (20.86% species‐level; 21.50% genus‐level) and tolerance (18.98% species‐level; 17.71% genus‐level), these dimensions appear to be the most important dimensions explaining the general pattern of the mammalian social organization. The FGMM revealed a total of three pronounced clusters, mostly determined by primary unit (Figure 2). All society components with individuals as their primary unit separated into one cluster. A second cluster comprised mostly unrelated larger groups with extended offspring membership philopatric patterns. A third cluster consisted mostly of pairs, families, and less stable groups. This partition into three clusters reflects Kappeler's description of forms of social organization for animals (Kappeler, 2018): live solitarily, coordinate with a partner (as a pair), or with a group. A large number of species in the first cluster with individuals as the primary unit suggest that there is a fourth option to this theory: a social lifestyle without coordinating all activities as a fixed group.

**Figure 3:**
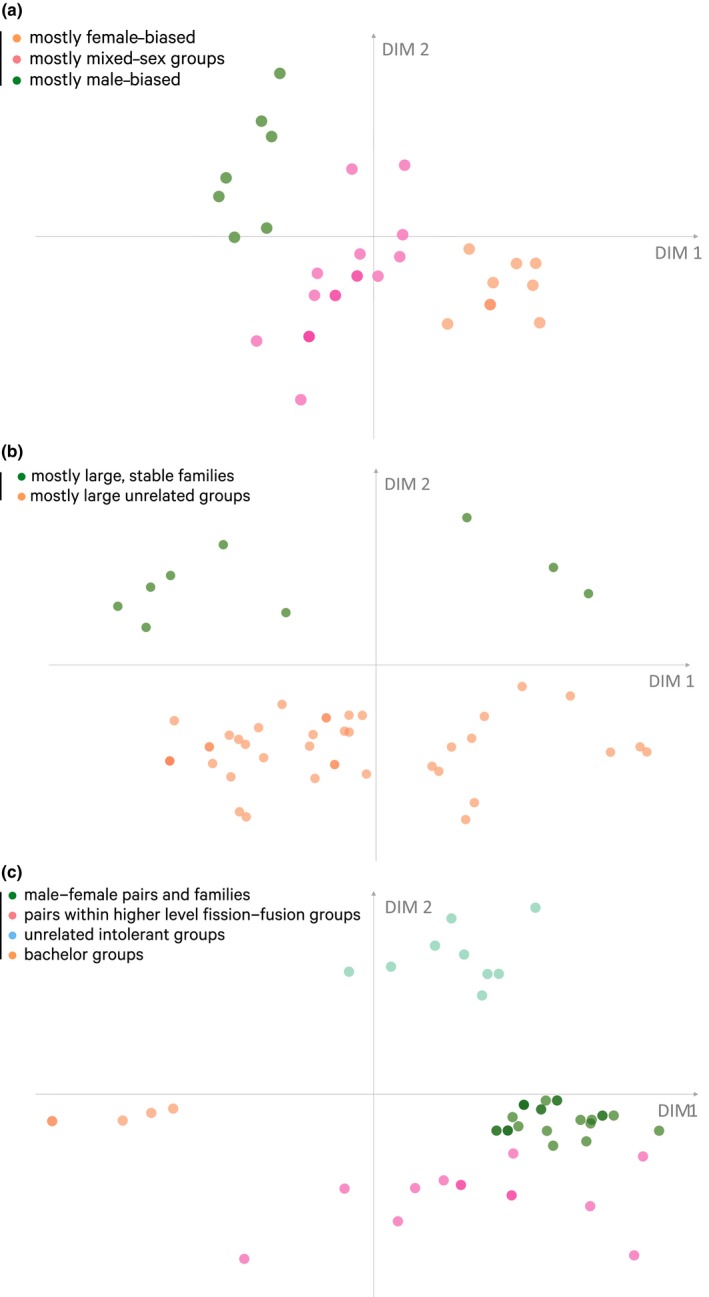
Distribution of society components across cluster landscape within clusters. Social components are positioned and colored using the same technique as Figure 2. (a) Patterns of subclusters from cluster 1: dark green: (seasonally persistent) male–female pairs and families; pink: pairs that form part of a higher level; light blue: unrelated intolerant groups; orange: “bachelor groups”: small unrelated groups of males; dark blue: large unrelated groups with no or short offspring membership. (b) Patterns of subclusters from cluster 2: green subcluster consists of mostly large, stable families with philopatric offspring; orange subcluster consists of mostly large unrelated groups. (c) Patterns of subclusters from cluster 3: the sex ratio of the society components within the orange subcluster is mostly female‐biased, the pink subcluster unbiased, and the green subcluster consists of social components with male‐biased sex ratios. The results from the corresponding genus‐level analyses are given in Figure A4

**Figure A2:**
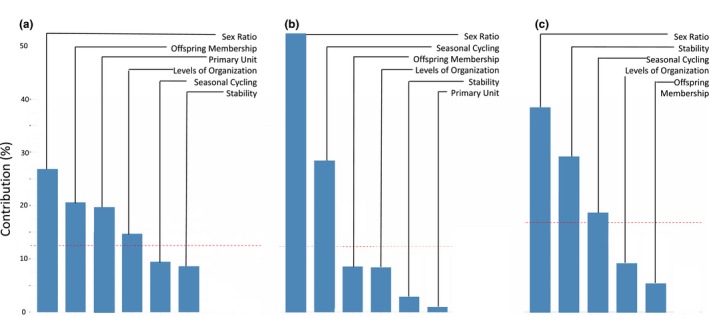
Contribution of the eight dimensions to first principal component within the three main clusters. The percentage is the contribution of data from that dimension that contributes to the first component after performing the PCA on the scores of the society components and then performing it a second time on the resulting clusters assigned by the FGMM. The red dashed line indicates the expected average contribution. (a) contribution of dimensions to first PC within cluster 1 (pairs, families, and less stable groups); (b) contribution of dimensions to first PC within cluster 2 (large stable groups); (c) contribution of dimensions to first PC within cluster 3 (groups with high degree of fission–fusion)

We repeated both the PCA and FGMM analysis within each cluster to identify finer‐scale partitions among society components. In all cases, dimensions were not correlated by more than 65%, but the dimensions with the greatest influence varied across the three clusters. Primary unit only had the highest loading in the first cluster (pairs and less stable groups), while sex ratio had a high loading in all three clusters (Figure A2). The second cluster (pairs, families, and less stable groups) divided into four subclusters (Figure 3a): seasonally persistent male–female pairs, pairs that form part of a higher level, “bachelor groups” (small unrelated groups of males), and unrelated intolerant groups. In the second cluster (large stable groups), one subcluster consisted of mostly large unrelated groups, while the other consisted of mostly large, stable families with philopatric offspring (Figure 3b). The third cluster (groups with high degree of fission–fusion) clustered into three subclusters that had a sex ratio mostly biased toward females, unbiased, and male‐biased (Figure 3c).

**Figure 4:**
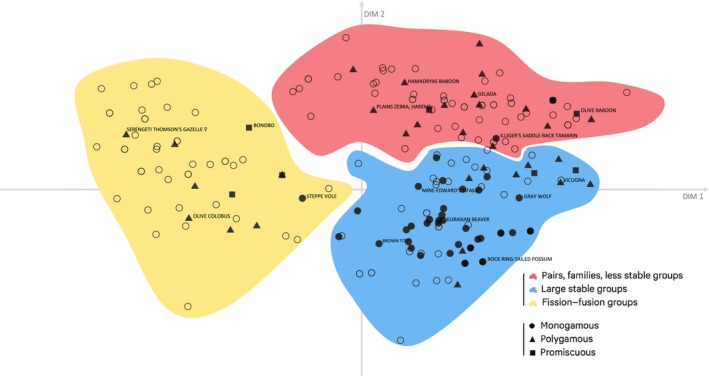
Distribution of different mating systems across cluster landscape. Only the three most common mating systems are displayed: monogamous (filled circles), polygynous (triangles), and promiscuous (squares)

**Figure 5:**
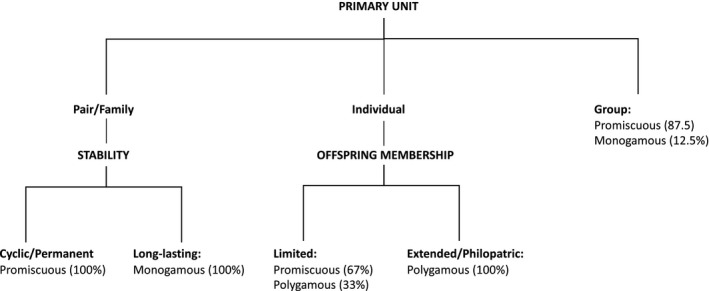
Decision tree resulting from a cross‐validation test. The outcomes of the correctly predicted mating systems are given in percentages

We then tested whether social organization (outside of the breeding season) was linked to mating systems. The DFA revealed an accuracy of 66.67%, which suggests that mating systems could be estimated fairly well using the information provided by the variables, but not entirely (Figure 4). The decision tree revealed that some of the dimensions are stronger predictors of mating systems, namely primary unit, stability, and offspring membership (Figure 5).

## 5 DISCUSSION

We developed framework that identified distinct attributes of social organization that can be used to better describe mammalian societies and facilitate comparisons across species. Based on our findings, the primary unit appears to be a key dimension for categorizing societies, underlying most of the variation across species. Although primary unit predominantly splits species into three distinct clusters, in line with previous studies (Kappeler, 2018), we found some evidence that the partitioning may require revisiting. Our framework also highlights that while mating systems are an important component of mammal sociality and certainly often the major cause of how societies form, they are not entirely linked to social organization. Social organization and mating systems appeared most strongly tied via the primary unit, whereas other dimensions were more independent from mating systems. Our study has shown that there are major new insights to be gained by studying social organization in greater detail, and we provide the necessary framework to do so.

Our framework is suitable for all mammal societies with few exceptions. A possible limitation is that some species' features might fall between the scores, for example, where species usually live in pairs but have been observed with more than one adult female. A possible solution to this would be to include the flexibility of social organization as an extra dimension. We did not do so in this framework, as data for this are rarely available. While it is fairly easy to rate a social system as flexible after encountering few varying social groups, the decision to describe a social system as inflexible needs to be supported by a large sample size (Papageorgiou et al., 2019). By contrast, the dimensions we outline are usually straight forward to describe with a good knowledge of the natural history of a species.

We found that primary unit, tolerance, and stability form the central parts of mammalian social organization. The intersection between these dimensions is highlighted by the suggestion of a potential fourth option for social organization (next to living solitarily, coordinating with a partner (as a pair), or with a group (Kappeler, 2018)) based on primary unit: social life without coordinating all activities as a fixed group. As the hierarchical clustering shows, primary unit, tolerance, and stability seem to be relatively independent of other dimensions, such as offspring membership, sex ratio, and group size. This is a critical finding as most comparative studies focused on social behavior, such as the link between brain size and social complexity (Dunbar, 1998), have used measures of group size as a metric for organizational complexity. Our framework instead suggests that primary unit may in fact be a better measure to capture large‐scale variation in social organization across species in situations requiring a single score (such as most comparative analyses).

**Figure 6:**
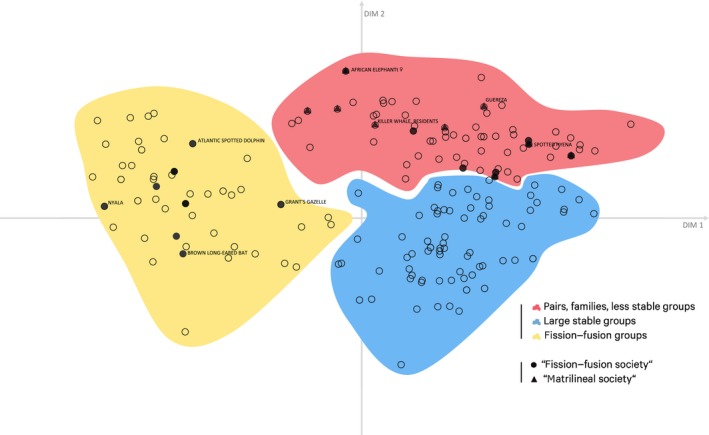
Distribution of fission–fusion and matrilineal societies across cluster landscape. Both fission–fusion and matriarch societies were scored as such, if this term was used in the chapter of the regarding species of the Handbook of the Mammals of the World (Wilson & Mittermeier, 2009). Filled circles indicate so‐called fission–fusion societies, and triangles represent matrilineal societies

The classification by primary unit might seem obvious, but when looking at terms used for different societies, it shows that societies are not always principally regarded in this way or rigidly structured by them. The most striking examples are so‐called fission–fusion societies that can be found all across the social organization landscape of the framework (Figure 6), mapping the term “fission–fusion” when used associated with species in HBMW (Wilson et al., 2019; ). However, if the term “fission–fusion society” is interpreted in a way where it refers to unpredictable group memberships with individuals as the primary unit, all of such society components would be found within the same cluster. Matrilineal systems, on the other hand, cluster together and show similarities beyond primary unit and patterns of dispersal. Mating system showed some patterning across clusters with monogamous systems predominating in the second cluster (pairs, families, and less stable groups).

As more species have dimensions of their social systems, comparing the way societies cluster into different groups and the importance of each dimension could help unpick drivers arising from environment and/or life histories. Further exploration should be conducted on when and how different features of social systems in mammals developed. If mating systems are not entirely explained by societies' social organizations, future work could be directed toward exploring the links between those two aspects of societies. Finally, the framework could be relatively easily extended to other taxa, allowing larger‐scale investigations into causes and consequences underlying the evolution of social organization.

Categorizing aspects of mammalian societies in a more precise and consistent framework can shed light on patterns that otherwise remain unclear. Looking at social organization separately from other aspects of sociality in mammals has enabled us to identify distinct underlying patterns that reflect other, seemingly equally important, aspect of animals' social lives. Putting these patterns into relation with other aspects of animal social systems, such as mating systems, suggested some consistencies as well as some differences—the latter meriting more investigation in the future. We hope that this framework will be an aid to further explore global social patterns and drawing comparisons across species.

## CONFLICT OF INTEREST

There are no competing interests related to this study.

## AUTHOR CONTRIBUTIONS

Lea Prox involved in the development of framework, performed statistical analysis, and wrote the manuscript. Damien Farine involved in the development of framework and supervised the study.

### Open Research Badges

This article has been awarded Open Data and Open Materials Badges. All materials and data are publicly accessible via the Open Science Framework at https://doi.org/10.6084/m9.figshare.8208959.v2 and https://doi.org/10.6084/m9.figshare.8208959.v2.

## Figures and Tables

**Figure A3:**
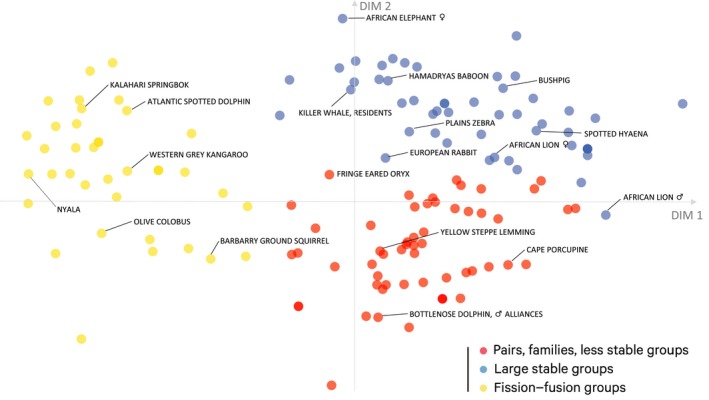
Distribution of society components across cluster landscape after reducing to one society component per genus. After performing a PCA, the resulting coordinates of 208 society components across 129 genera for each of the eight variables were reduced to two dimensions. The circles represent the society components and have been colored according to the clusters they were assigned to by the FGMM. Society components within the yellow cluster are characterized by “individual” as primary unit, fluid stability, and mostly open tolerance. Society components within the blue cluster are mostly larger groups and families with long‐lasting to philopatric offspring membership and long‐lasting to permanent stability. The red cluster consists of society components that mostly live in pairs or families with offspring that are generally not philopatric (except for few exceptions). Society components tend to be more tolerant within the red cluster than the blue

**Figure A4:**
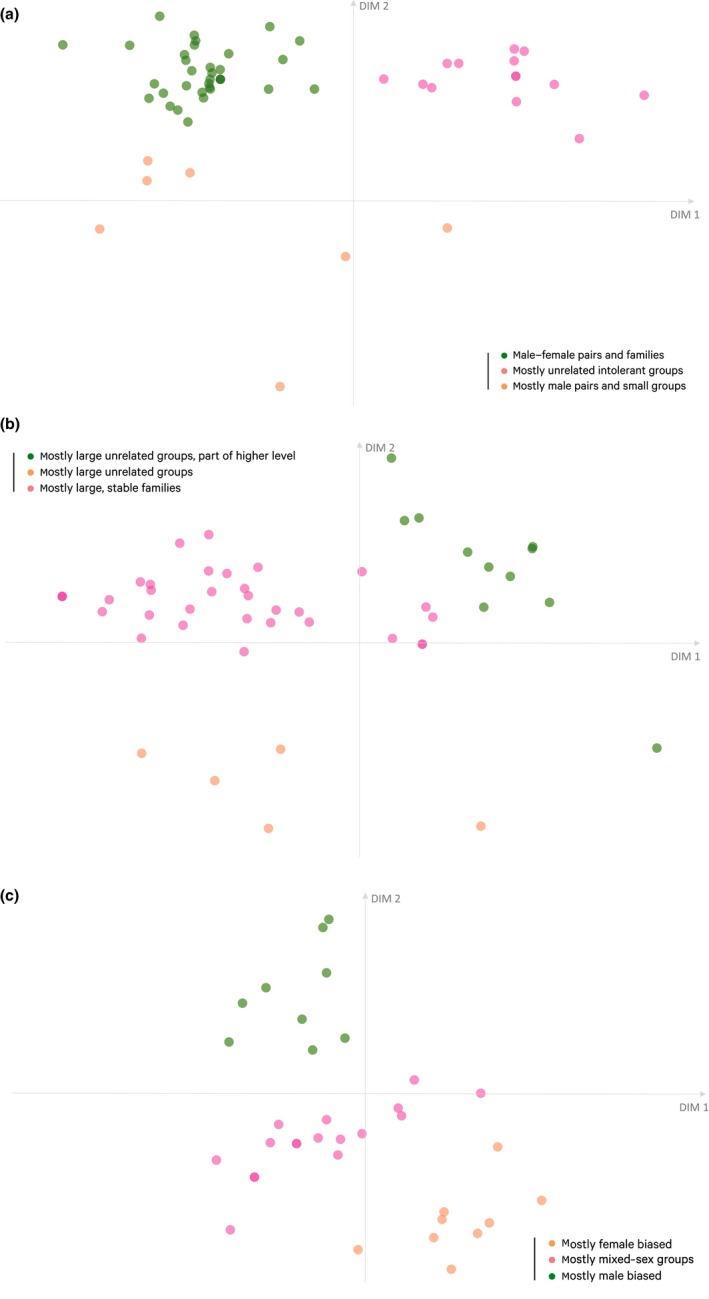
Distribution of society components across cluster landscape within clusters after reducing to one society component per genus. Social components are positioned and colored using the same technique as Figure 2. (a) Patterns of subclusters from cluster 1: dark green: (seasonally persistent) male–female pairs and families; pink: mostly unrelated intolerant groups; orange: mostly male pairs and small groups (b) Patterns of subclusters from cluster 2: green subcluster consists of mostly large, stable families with philopatric offspring; orange subcluster consists of mostly large unrelated groups; pink subcluster: mostly large, stable families (c) Patterns of subclusters from cluster 3: the sex ratio of the society components within the orange subcluster is mostly female‐biased; pink subcluster mixed‐sex groups, and the green subcluster consists of social components with male‐biased sex ratios

## Data Availability

Data used for this paper and scripts are available on Figshare: https://doi.org/10.6084/m9.figshare.8208959.v2.
